# Targeting density-enhanced phosphatase-1 (DEP-1) with antisense oligonucleotides improves the metabolic phenotype in high-fat diet-fed mice

**DOI:** 10.1186/1478-811X-11-49

**Published:** 2013-07-26

**Authors:** Janine Krüger, Manuela Trappiel, Markus Dagnell, Philipp Stawowy, Heike Meyborg, Christian Böhm, Sanjay Bhanot, Arne Östman, Ulrich Kintscher, Kai Kappert

**Affiliations:** 1 Center for Cardiovascular Research/CCR, and Institute of Laboratory Medicine, Clinical Chemistry and Pathobiochemistry, Charité–Universitätsmedizin, Berlin, Germany; 2 Cancer Center Karolinska, Karolinska Institutet, Stockholm, Sweden; 3 Department of Medicine/Cardiology, Deutsches Herzzentrum, Berlin, Germany; 4 Center for Cardiovascular Research/CCR, and Institute of Pharmacology, Charité–Universitätsmedizin, Berlin, Germany; 5 ISIS Pharmaceuticals, Inc, Carlsbad, CA, USA

**Keywords:** Protein-tyrosine-phosphatase, Density-enhanced phosphatase-1, Insulin resistance, Type 2 diabetes, Antisense oligonucleotides, Metabolic tissues, Insulin signaling, Insulin receptor, Obesity

## Abstract

**Background:**

Insulin signaling is tightly controlled by tyrosine dephosphorylation of the insulin receptor through protein-tyrosine-phosphatases (PTPs). DEP-1 is a PTP dephosphorylating tyrosine residues in a variety of receptor tyrosine kinases. Here, we analyzed whether DEP-1 activity is differentially regulated in liver, skeletal muscle and adipose tissue under high-fat diet (HFD), examined the role of DEP-1 in insulin resistance *in vivo*, and its function in insulin signaling.

**Results:**

Mice were fed an HFD for 10 weeks to induce obesity-associated insulin resistance. Thereafter, HFD mice were subjected to systemic administration of specific antisense oligonucleotides (ASOs), highly accumulating in hepatic tissue, against DEP-1 or control ASOs. Targeting DEP-1 led to improvement of insulin sensitivity, reduced basal glucose level, and significant reduction of body weight. This was accompanied by lower insulin and leptin serum levels. Suppression of DEP-1 *in vivo* also induced hyperphosphorylation in the insulin signaling cascade of the liver. Moreover, DEP-1 physically associated with the insulin receptor *in situ*, and recombinant DEP-1 dephosphorylated the insulin receptor *in vitro*.

**Conclusions:**

These results indicate that DEP-1 acts as an endogenous antagonist of the insulin receptor, and downregulation of DEP-1 results in an improvement of insulin sensitivity. DEP-1 may therefore represent a novel target for attenuation of metabolic diseases.

## Lay abstract

Insulin resistance represents a main factor contributing to type 2 diabetes in obese patients. The tremendous increase of type 2 diabetes has developed to a world-wide epidemic burden. However, the cellular mechanisms underlying insulin resistance are only partly understood. Therefore, a better understanding of the pathophysiology and the molecular background of insulin resistance are highly warranted. Several studies have described an increased protein-tyrosine-phosphatase activity in metabolic tissues in obesity. Indeed, certain protein-tyrosine-phosphatases are known to target the insulin receptor and negatively regulating the insulin signaling pathway. We observed that the activity of density-enhanced phosphatase-1 (DEP-1), a receptor-like transmembrane protein-tyrosine-phosphatase, is upregulated in obese insulin resistant mice. Additional studies showed the capacity of DEP-1 to dephosphorylate the insulin receptor. Furthermore, reducing DEP-1 by a pharmacological approach in mice improved insulin sensitivity, reduced basal glucose level, and led to lower body weight. Moreover, as shown in liver tissues, DEP-1 physically associated with the insulin receptor. Taken together, this study identifies the phosphatase DEP-1 as novel component in insulin signaling, and molecular target for the treatment of insulin resistance and obesity-associated diseases.

## Background

Obesity represents a significant health problem with epidemic proportions worldwide. Obesity-related insulin resistance is fundamentally linked to development of type 2 diabetes, with critical impact on hypertension, atherosclerosis, and hyperlipidemia [1]. Thus, novel treatment regimens are desired to face this enormous health burden, and to decrease morbidity/mortality in insulin resistant obese patients.

Insulin resistance is characterized by reduced glucose uptake, metabolism, or storage, and impaired suppression of hepatic glucose output. The activity of the insulin receptor kinase, a receptor tyrosine kinase (RTK), is determined by the phosphorylation status, and is tightly regulated by protein-tyrosine-phosphatases (PTPs) [2]. Thereby, PTPs inhibit postreceptor signaling in insulin-responsive tissues such as adipose tissue, muscle, and the liver. So far, 38 so-called “classical PTPs” have been identified in the human genome, which all share a catalytic signature motif V/I H C S X G. These PTPs represent one subgroup of phosphatases with strict tyrosine-specificity [3]. PTP activity has been described to be dynamically enhanced in obesity in insulin-sensitive tissue (adipose tissue, skeletal muscle and liver) [4], with significant reduction after weight loss [5,6]. So far, various PTPs were identified targeting the insulin receptor kinase: PTP1B, SHP-1, SHP-2, CD45, LAR, PTPalpha, and PTPepsilon [7-9]. Thus, efforts have been undertaken to dissect the role of PTPs in insulin signaling and metabolic diseases.

In particular, PTP1B has been studied as potential therapeutic drug target in obesity and insulin resistance, since genetic interruption resulted in resistance to high-fat diet-induced insulin resistance and obesity [9,10]. Those PTP1B-deficient mice were characterized by increased phosphorylation of the insulin receptor in both liver and muscle tissue upon insulin challenge, as compared to wild-type mice [9]. Furthermore, *in vitro* data demonstrated direct interaction of PTP1B with the insulin receptor, leading to efficient dephosphorylation of tyrosine residues [9,11]. In contrast, PTP1B inhibition enhances insulin receptor signals [12,13]. Type 2 diabetic individuals have recently been shown to have dysregulated PTP1B gene expression in the skeletal muscle [2], giving evidence that PTP1B is also critically involved in human pathology. Besides PTP1B, SHP-1 has attracted attention, since SHP-1 deficient mice were characterized by improved insulin receptor signaling to insulin receptor substrate-PI3K-Akt in liver and muscle [8]. Furthermore, inhibition of SHP-1 via adenoviral gene transfer resulted in enhanced insulin receptor tyrosine- as well as Akt (at serine 473) phosphorylation in myocytes upon insulin stimulation [14]. Thus, PTP inhibition might constitute a useful approach for treatment/prevention of obesity-associated insulin resistance and type 2 diabetes. However, with regard to PTP1B, development of efficient antagonists has been hampered by a variety of factors, including low selectivity and bioavailability [15]. Antisense oligonucleotides (ASOs) could overcome this burden and were shown to be effective in both rodents and primates [13,16,17].

The density-enhanced phosphatase (DEP)-1 was initially described to contribute to the mechanism of contact inhibition of cell growth [18]. Moreover, DEP-1 is upregulated by protective nutrients [19], and plays a pivotal role in determining neointima formation upon vascular injury [20]. It was shown that DEP-1 interacts with a variety of RTKs, including the platelet-derived growth factor (PDGF) receptor beta [21], and the hepatocyte growth factor (HGF) receptor c-Met [22]. A potential role of DEP-1 in insulin receptor signaling has not been described. Here we speculated that, based on its binding to various tyrosine residues in RTKs, DEP-1 may directly or indirectly interfere with insulin receptor signaling. First hints for such an involvement of DEP-1 were given by positive dephosphorylating effects using an 18-amino acid phosphopeptide corresponding to three insulin receptor kinase autophoshorylation sites using the catalytic domain of DEP-1 [23]. Thus, the present study was done to elucidate the role of DEP-1 in insulin signaling, including its potential binding to the tyrosine phosphorylated insulin receptor, and to investigate the effects of ASOs targeting DEP-1 (ISIS 285564) in a metabolic high-fat diet-induced obesity model characterized by reduced insulin sensitivity.

## Results

### DEP-1 activity is increased in high-fat diet-induced obesity

**Figure 1:**
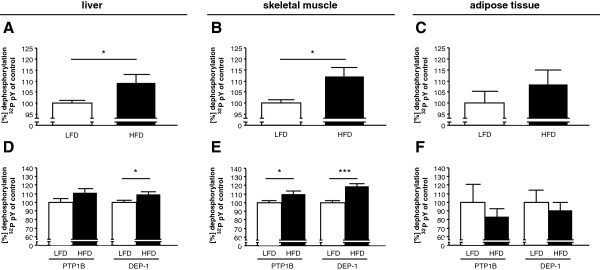
**Diet-induced obesity increased pan-PTP activity and DEP-1 activity in liver and skeletal muscle in mice. A-C**: Pan-PTP activity was determined in liver, skeletal muscle and adipose tissue by measuring dephosphorylation of a ^32^P labeled phosphopeptide in total lysates from mice fed an LFD or HFD for 16 weeks. Pan-PTP activity in mice fed with LFD were set to 100%; (n = 10 per group). **D-F**: Dephosphorylation of a ^32^P labeled phosphopeptide was measured for precipitated PTP1B and DEP-1 in LFD and HFD mice. Tissue lysates were subjected to immunoprecipitation. PTP1B and DEP-1 activity in mice fed with LFD were set to 100%; (n = 5–6 per group). *P < 0.05, ***P < 0.001.

The tyrosine-phosphatase activity – pan-PTP activity – in insulin sensitive tissues was analyzed in mice fed with an LFD or HFD for 16 weeks. HFD mice exhibited a significant increase in body weight (LFD = 28.8 ± 0.8 g vs. HFD = 32.2 ± 0.5 g; *P* < 0.01). Based on previous data showing differential regulation of PTPs in models of obesity/insulin resistance, we analyzed PTP activity in mice subjected to LFD or HFD. A significant increase of pan-PTP activity was detected in liver (Figure 1A) and skeletal muscle (Figure 1B) in HFD mice. Also in adipose tissue pan-PTP activity was higher in HFD mice than in LFD mice (Figure 1C), but did not reach statistical significance. To dissect which individual PTPs were responsible for the increase of pan-PTP activity after HFD feeding we determined the activity of specific PTPs after immunoprecipitation. PTP1B, described as a negative regulator in insulin signaling [9,10] and previously demonstrated increased activity under HFD in metabolic tissues [24], was compared to DEP-1 activity. Measurements of the specific DEP-1 activity revealed a significant upregulation in liver (Figure 1D) and skeletal muscle (Figure 1E) under HFD. However, in the adipose tissue no increase in PTP1B- and DEP-1 activity was detected in HFD mice (Figure 1F). These results provide evidence that DEP-1 is upregulated in diet-induced obesity.

### Reduction of DEP-1 expression and DEP-1 activity by ASOs in high-fat diet-induced insulin resistant mice

**Figure 2:**
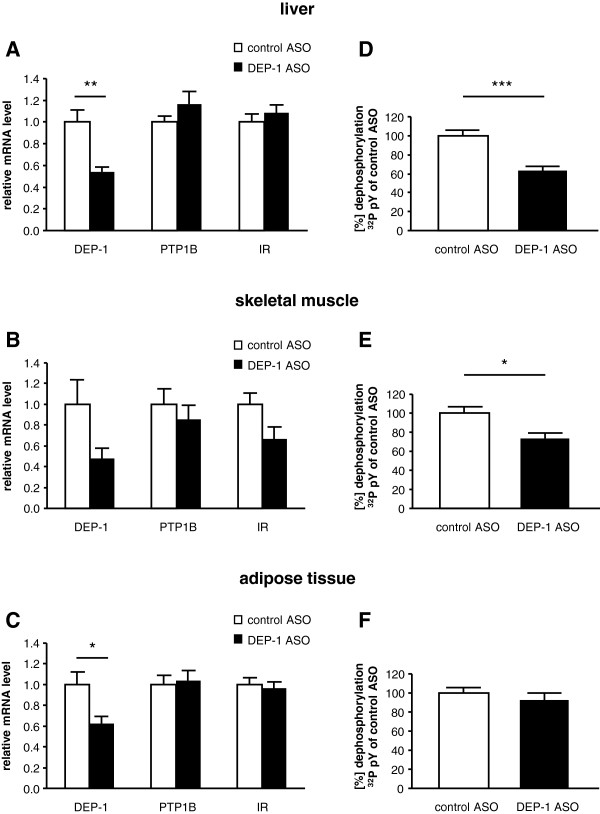
**Administration of DEP-1 antisense oligonucleotides (ASOs) reduced DEP-1 expression and activity in metabolic tissues. A-C**: Quantitative real-time PCR was performed for DEP-1, PTP1B and insulin receptor (IR) in liver, skeletal muscle and adipose tissue mRNA from mice subjected to either control ASO or DEP-1 ASO treatment. Gene expression was normalized to expression of the housekeeping gene *18S* and is shown as mean ± standard error of the mean; (n = 8–9 per group). **D-F**: DEP-1 activity was measured using a dephosphorylation assay of a ^32^P labeled phosphopeptide after immunoprecipitation of DEP-1 in liver, skeletal muscle and adipose tissue from mice subjected to either control ASO or DEP-1 ASO treatment. DEP-1 activity in control ASO mice were set to 100%; (n = 6 per group). *P < 0.05, **P < 0.01, ***P < 0.001.

Based on the increase in DEP-1 activity in diet-induced obesity (Figure 1D-E) – measured under reduced conditions – we hypothesized that DEP-1 plays a role in metabolic changes and insulin signaling. Thus, HFD-fed mice were treated with antisense oligonucleotides (ASOs) against DEP-1 or control ASOs. To confirm successful ASO application and subsequent attenuation of DEP-1 we analyzed the extent of DEP-1 suppression on transcript level and DEP-1 activity. In HFD-fed mice treated with ASOs targeting DEP-1, a significant reduction of mRNA levels by ~46% in the liver (Figure 2A), and by ~38% in adipose tissue (Figure 2C) was observed compared to control ASO mice. Also in skeletal muscle a nonsignificant suppression was observed (Figure 2B). However, “secondary effects” may explain this unexpected reduction in transcripts in skeletal muscle, since ASOs are not considered to significantly distribute to skeletal muscle tissue regulating target genes [25]. Analyzing PTP1B mRNA levels revealed no compensatory regulation, and also insulin receptor mRNA was not affected by DEP-1 suppression in all tissues. Furthermore, DEP-1 activity was significantly decreased in liver (Figure 2D) and skeletal muscle (Figure 2E) after DEP-1 ASO treatment, while, surprisingly, no reduction of DEP-1 activity was measured in adipose tissue (Figure 2F) compared to control ASO mice.

**Figure 3:**
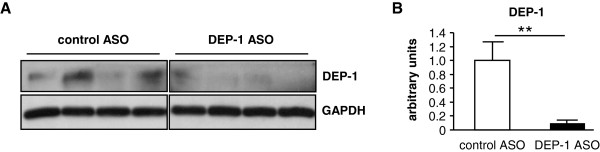
**DEP-1 protein expression is reduced in the liver after antisense oligonucleotide (ASO) treatment. A**: DEP-1 protein expression was analyzed after wheat-germ-agglutinin (WGA) precipitation and visualized by immunoblotting of tissues derived from control ASO and DEP-1 ASO treated mice; (n = 4 per group). **B**: Densitometric analysis of immunoblotting data was performed after normalization to the housekeeping protein GAPDH and expressed as arbitrary units; (n = 8–9 per group). **P < 0.01.

In addition to DEP-1 mRNA transcription level and DEP-1 activity we analyzed DEP-1 protein expression in the liver by immunoblotting analysis (Figure 3A). We detected a significant downregulation of DEP-1 in the DEP-1 ASO group compared to control ASO treated mice (Figure 3B), substantiating the DEP-1 mRNA and DEP-1 activity measurements.

To exclude ASO effects *per se* on total tyrosine phosphorylation levels we performed immunoblotting in liver tissue derived from ASO-treated and untreated mice (Additional file 1: Figure S1). This analysis did not show changes in liver tyrosine phosphorylation due to ASO treatment.

Summarized, DEP-1 ASO administration resulted in an effective reduction of DEP-1 transcripts, activity and protein expression in liver of HFD-mice.

### DEP-1 suppression improves metabolic parameters in high-fat diet-treated mice

**Figure 4:**
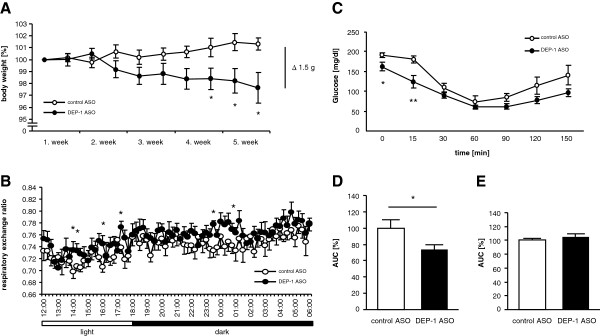
**Basic metabolic phenotyping of mice subjected to control ASO and DEP-1 ASO administration. A**: Body weight was monitored twice weekly during the application period with ASOs; (n = 8–10 per group). **B**: Respiratory exchange ratio (RER) was measured over 18 h in metabolic cages (LabMaster). Each data point represents n = 8–9 animals per group. **C**: An intraperitoneal insulin tolerance test (ITT) was performed 5 weeks after ASO administration by injection of 0.5 U insulin/kg body weight to fasted mice; (n = 7 per group). **D**: Corresponding AUC to (C). **E**: After 6 weeks of ASO administration a glucose tolerance test (GTT) was performed in fasted mice by injection of 1 g glucose/kg body weight quantified by AUC; (n = 8–9 per group). *P < 0.05, **P < 0.01.

During the application period the body weight of control ASO and DEP-1 ASO treated mice under HFD were repetitively determined, and a time-dependent significant reduction was observed in DEP-1 ASO animals when compared to control ASO (Figure 4A). After five weeks of ASO treatment the body weight difference between both groups was 1.5 g (control ASO 34.0 ± 0.7 g and DEP-1 ASO 32.5 ± 0.5 g; *P* < 0.05), and independent of mean food intake (Table 1). Additionally, in congruence with the body weight loss, the weight of epididymal fat as well as perirenal fat was significantly reduced in DEP-1 ASO treated mice, along with increased liver weight (Table 1), which is in line with previous observations in PTP1B ASO mice [4,17]. In contrast, kidney and heart weight remained unchanged.

**Table 1:** Metabolic phenotyping of control and DEP-1 ASO treated mice

	**Control ASO**	**DEP-1 ASO**	***P-value***
**LabMaster analysis**			
Food intake [g]	1.7 ± 0.2	2.0 ± 0.1	0.230
Water intake [ml]	1.8 ± 0.2	2.1 ± 0.2	0.317
Temperature [°C]	20.379 ± 0.015	20.520 ± 0.018	< 0.001
Locomotor activity [x + y]	61426 ± 5246	53600 ± 4465	0.287
Locomotor activity [z]	6296 ± 1095	4678 ± 770	0.217
**Organ weight**			
**[organ weight mg/body weight g]**			
Epididymal fat	18.4 ± 1.7	13.8 ± 1.0	0.038
Perirenal fat	7.3 ± 0.8	5.1 ± 0.6	0.048
Liver	38.6 ± 1.7	51.8 ± 0.9	< 0.001
Heart	4.8 ± 0.3	4.3 ± 0.1	0.197
Kidney	11.3 ± 0.9	11.1 ± 0.5	0.830

Control ASO and DEP-1 ASO mice were characterized using metabolic cages (LabMaster) 4 weeks after ASO treatment. Measurements of an 18 hours period (12 am–6 am) are shown. Data were recorded every 15 min. After sacrificing of mice, organs weights were determined. Data are shown as mean ± standard error of the mean.

Further, we analyzed whether energy metabolism parameters were affected in DEP-1 ASO mice by monitoring animals over 18 hours in metabolic cages (LabMaster), measuring food intake, water intake, cage temperature, locomotor activity (Table 1) as well as respiratory exchange ratio (RER) (Table 1 and Figure 4B). A significant difference in RER between both groups was detected, indicating a shift to carbohydrate utilization in DEP-1 ASO treated mice. Furthermore, cage temperature was significantly higher in the DEP-1 ASO group, suggesting an impact on thermogenesis. No significant difference between DEP-1 ASO and control ASO treated mice was detected in locomotor activity.

**Figure 5:**
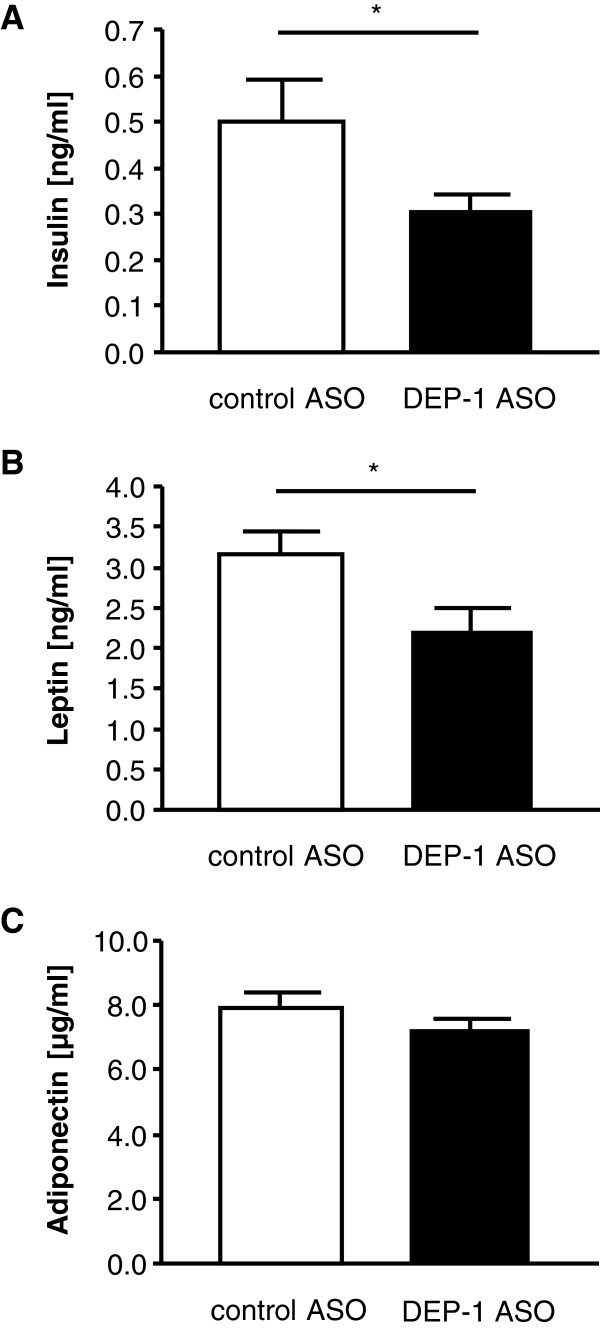
**DEP-1 ASO mice were characterized by improvement in metabolic serum parameters.** Fasting serum insulin **(A)**, leptin **(B)**, and adiponectin levels **(C)** were determined by ELISA measurements; (n = 6–9 per group). *P < 0.05.

To address whether DEP-1 suppression led to improvement of insulin and glucose tolerance mice were subjected to both intraperitoneal ITT and GTT. DEP-1 ASO treated mice showed improvement in insulin sensitivity, with significant reduction in glucose levels at baseline and 15 min after insulin injection compared to control ASO mice (Figure 4C), also evidenced when the area under the curve (AUC) was calculated (Figure 4D). However, DEP-1 ASO treatment did not result in an improved glucose tolerance (Figure 4E). In addition to the improved insulin sensitivity, DEP-1 ASO treated mice also showed significantly decreased fasting insulin and leptin levels compared to control ASO mice (Figure 5A-B). In contrast, adiponectin concentrations were not altered by DEP-1 ASO treatment (Figure 5C).

### DEP-1 suppression leads to increased phosphorylation levels in insulin signaling in vivo

**Figure 6:**
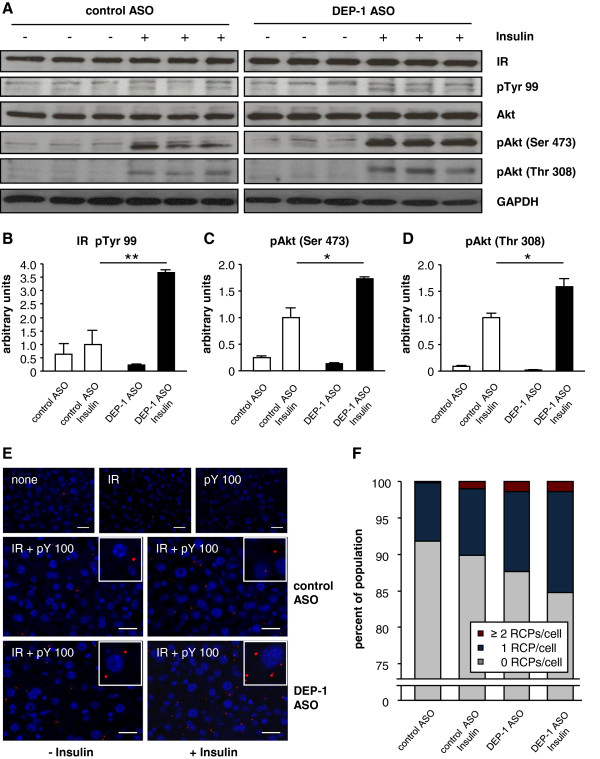
**Insulin signaling was enhanced in DEP-1 ASO mice. A**: Immunoblotting analysis of liver lysates in control ASO or DEP-1 ASO treated mice challenged with intraveneous injection of insulin (10 U/kg body weight, 2 min) prior to euthanasia; (n = 3 per group). Key intermediates of the insulin signaling cascade were analyzed with indicated antibodies. GAPDH was used as the loading control. **B**-**D**: Densitometric analysis of immunoblotting for pTyr 99 (normalization to IR), pAkt (Ser 473) and pAkt (Thr 308) (normalization to Akt) were performed and expressed as arbitrary units; (n = 3 per group). *P < 0.05, **P < 0.01. **E**: Detection of phosphorylated insulin receptor in liver tissue of mice. Insulin receptor tyrosine phosphorylation was detected by *in situ* PLA (*red dots*) applying anti-insulin receptor and anti-phosphotyrosine antibodies. Liver tissue sections derived from C57BL/6J mice subjected to either control ASOs or DEP-1 ASOs and insulin stimulation, as described in **A**. The cells were counterstained with DAPI (blue) to visualize the nuclei. Specificity of antibody binding and PLA method is demonstrated by control sections with either only antibodies against the insulin receptor (IR), against phosphotyrosine (pY100), or omitting primary antibodies (none). Insets of representative cells are shown. Scale bars represent 25 μm. **F**: The quantification of PLA signals per cell for animal groups is depicted as the percentage of each population with a certain number of RCPs per cell (n = 424–458 cells per group).

Phosphorylation levels of key intermediates in insulin signaling were analyzed in metabolic tissues by immunoblotting to investigate how DEP-1 suppression modulates this signaling pathway. Analyses of control ASO and DEP-1 ASO treated mice were done in animals which were challenged with or without intraveneous injection of insulin 2 min prior of sacrificing. In liver tissues an increase of total tyrosine phosphorylation of the insulin receptor after insulin administration was observed in the DEP-1 ASO group compared to control ASO mice (Figure 6A). Furthermore, the phosphorylation of Akt at sites Thr 308 and Ser 473 were increased in DEP-1 ASO mice (Figure 6A) compared to control ASO mice, suggesting further impact on downstream signaling by DEP-1 suppression. In addition, densitometric analysis of insulin receptor phosphorylation and Akt phosphorylation at sites Thr 308 and Ser 473 revealed a significant increase after insulin challenge in DEP-1 ASO treated mice (Figure 6B-D). Phosphorylation levels of insulin signaling intermediates in adipose tissue were equal in both groups (data not shown), which is in accordance that DEP-1 activity was unaffected by DEP-1 ASO treatment (Figure 2F). To substantiate the finding of higher insulin receptor phosphorylation in DEP-1 ASO-treated mice, liver sections were subjected to Proximity Ligation Assays (PLA) using co-incubation of antibodies directed against the insulin receptor and phosphotyrosine residues. Representative sections are depicted in Figure 6E, demonstrating higher hepatic *in situ* insulin receptor tyrosine phosphorylation in DEP-1 ASO treated mice, as compared to that observed in insulin-treated control ASO mice, which is also demonstrated by a cell based quantification in Figure 6F.

### DEP-1 is recruited to the insulin receptor upon insulin stimulation in situ

**Figure 7:**
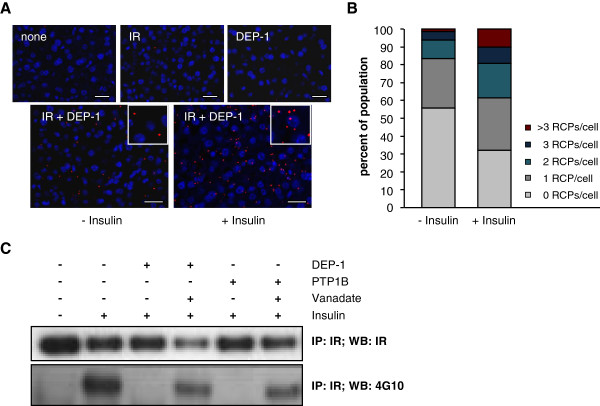
**Detection of the recruitment of DEP-1 to the insulin receptor. A**: The recruitment of DEP-1 to the insulin receptor was detected by using *in situ* PLA (red dots) in liver tissue sections of C57BL/6J mice which were either untreated or stimulated with insulin (10 U/kg body weight, 2 min). The cells were counterstained with DAPI (blue) to visualize the nuclei. Insets of representative cells are shown. Scale bars represent 25 μm; none, no primary antibody; insulin receptor (IR), rabbit anti-IR antibody; DEP-1, goat anti-DEP-1 antibody. **B**: The quantification of PLA signals per cell for both groups is depicted as the percentage of each population with a certain number of RCPs per cell (n = 350–500 cells). **C**: AML12 liver cells were used for evaluation of insulin receptor dephosphorylation by PTPs. Starved cells were left resting (−) or were stimulated with 100 nM insulin (+) for 10 min, followed by cell lysis and protein isolation. The immunoprecipitated insulin receptor was subjected to recombinant DEP-1 or PTP1B, as indicated, with or without preincubation with sodium vanadate (n = 3 experiments). Immunoblotting was performed with depicted antibodies.

Finally, after showing that DEP-1 was differentially regulated in metabolic tissues in diet-induced obesity, and that DEP-1 significantly impacts on insulin signaling, we addressed whether DEP-1 physically associates with the insulin receptor by recruitment studies applying PLA. This *in situ* method is able to visualize protein interactions at a resolution of ~40–50 nm. Figure 7A-B depicts representative pictures of liver sections, and a cell-based quantification, clearly demonstrating that DEP-1 is recruited to close proximity of the insulin receptor upon insulin stimulation.

Thus, these data support the notion that DEP-1 is a negative regulator of insulin signaling at the level of the insulin receptor, and may represent a novel target in insulin resistance.

### DEP-1 dephosphorylates the insulin receptor

To substantiate the findings of DEP-1 recruitment to the insulin receptor and hyperphosphorylation of the insulin receptor upon DEP-1 ASO administration, we analyzed the dephosphorylating capacity of DEP-1. AML12 liver cells were stimulated with insulin and the insulin receptor was subsequently immunoprecipitated following by incubation with recombinant DEP-1. As shown in Figure 7C, insulin-induced phosphorylation of the insulin receptor at tyrosine residues was clearly reduced by recombinant DEP-1. The dephosphorylation by DEP-1 was comparable to PTP1B, a phosphatase known to target and dephosphorylate the insulin receptor. Furthermore, inhibiting the phosphatase activity of both DEP-1 and PTP1B restored the tyrosine phosphorylation level of the insulin receptor. Taken together, DEP-1 exhibits insulin receptor dephosphorylation capacity.

## Discussion

In this study we demonstrate for the first time that the protein-tyrosine-phosphatase (PTP) DEP-1 is differentially regulated in a model of high-fat diet (HFD) induced obese mice, and that targeting DEP-1 leads to improvement of the metabolic phenotype.

In detail, we report that the activity of DEP-1 is increased in liver and skeletal muscle in HFD induced obese mice. To further analyze the role of DEP-1 in diet-induced obesity and in insulin signaling we applied DEP-1 antisense oligonucleotides (ASOs). This therapeutic approach with ASOs given to mice with reduced insulin sensitivity, resulted in an altered metabolic phenotype including reduced body weight, improved insulin sensitivity, and higher respiratory exchange ratio along with enhanced insulin signaling in liver. Moreover, we show that the insulin receptor is being dephosphorylated by recombinant DEP-1, and that DEP-1 is recruited into close proximity of the insulin receptor upon receptor ligation.

The activity of the PTPs PTP1B and SHP-1 [4,12] was earlier shown to be increased in obesity states in mice models. While DEP-1 was not previously described, the current study shows increased DEP-1 activity in both liver and skeletal muscle in obese animals. DEP-1 represents an ubiquitously expressed PTP interacting with various receptor tyrosine kinases like EGF receptor [26], HGF receptor [22], PDGF receptor [21], RET receptor [27], and VEGF receptor-2 [28]. DEP-1 is also involved in modulation of distinct key cellular components p85 [29], Akt/PKB [30] and Erk 1/2 [31], which are part of the insulin signaling pathway. Thus, this phosphatase may represent a therapeutic target of HFD-induced obesity and metabolic disorders including insulin resistance.

As expected, and shown earlier for other targets [16,17,32], in our study administration of DEP-1 ASOs lead to a significant reduction on DEP-1 transcript level in liver and adipose tissue in mice fed an HFD. Furthermore, we also observed a nonsignificant reduction of DEP-1 mRNA in the skeletal muscle correlating with a decrease of DEP-1 activity in mice subjected to DEP-1 ASO treatment. Indirect effects may be responsible for this unexpected result, based on changes in serum parameters and metabolism metabolites, which may impact on DEP-1 transcript levels leading to decreased DEP-1 activity in the skeletal muscle.

Furthermore, DEP-1 transcript levels were significantly reduced in adipose tissue, which did not translate into reduced DEP-1 activity. This was in line with findings that phosphorylation of key intermediates in insulin signaling was unaffected (data not shown). Interestingly, Takahashi et al. [33] recently reported binding of the newly identified DEP-1 ligand. Thrombospondin-1 was leading to increased DEP-1 catalytic activity and consecutively to dephosphorylation of substrate proteins. Thrombospondin-1, an adipokine shown to be increased in adipose tissue [34], might counteract the reduced DEP-1 transcript levels. Possibly, also inflammation factors secreted from adipose tissue affect the expression/activity of DEP-1, as shown earlier for PTP1B [24].

In addition to detection of lower DEP-1 transcript levels and DEP-1 activity (determined under reduced conditions), we performed immunoblotting analyses of precipitated DEP-1. Our data clearly demonstrate both efficiency and specificity of DEP-1 ASO treatment, since expression of DEP-1 was reduced while gene expression of other insulin signaling components was unchanged (PTP1B, insulin receptor) in epididymal tissue, skeletal muscle, and liver. Also additional PTPs (LAR, TC-PTP, SHP-2) were not regulated in liver tissue (data not shown), underlining specificity of the DEP-1 ASO used in our study. Furthermore, ASO treatment itself seems to have a neglectable impact *per se*, since no significant changes were detectable in total tyrosine phosphorylation patterns in liver tissues derived from untreated (HFD) and HFD-fed ASO-treated mice.

In general, DEP-1 ASO application leads to decreased endogenous mRNA level and therefore reduced protein and activity levels were detected, as we could show for the liver tissue.

Metabolic phenotyping revealed changes in body composition, evidenced by a significant decrease in body weight in mice receiving DEP-1 ASOs, along with a decrease in white fat pad mass in sacrificed mice. This lean phenotype was associated with an increase in the respiratory exchange ratio (RER). We and others have reported low RER in diabetic rodents and under HFD [35,36]. Both control ASO and DEP-1 ASO mice exhibited low RER values. However, HFD-treated mice subjected to DEP-1 ASO treatment were characterized by significantly higher RER values at defined time points. These data indicate augmented food metabolism towards higher ratio of carbohydrate utilization. However, further studies should focus on delineation of the underlying mechanism of increased RER in combination of reduced adipose tissue mass under HFD and PTP targeting through ASOs.

In the current study we were able to show that DEP-1 ASO treatment improved insulin sensitivity. This is in line with previous reports on ASO treatment targeting PTP1B and LMW-PTP in rodent models, which lead to beneficial impact on ITT [16,17]. The impact of DEP-1 ASOs on DEP-1 activity was only pronounced in liver and skeletal muscle compared to adipose tissue. In contrast to the effects on insulin sensitivity, DEP-1 ASO application did not produce detectable changes in glucose tolerance. This may favor the hypothesis that DEP-1 action in liver is more important than skeletal muscle effects, since glucose excursion in GTT is mainly mediated by the skeletal muscle. This might partly explain the apparent diverse effects in insulin and glucose tolerance.

The increased insulin sensitivity was accompanied by decreased insulin levels after DEP-1 ASO treatment. Further, leptin, an adipocyte secreted hormone, known to be increased in HFD with concordant regulation of adipose tissue growth [37], was reduced by DEP-1 ASOs, reflecting the observed lower body fat accumulation and decreased body weight. Adiponectin, a key player in regulation of insulin sensitivity, was shown to be upregulated after weight loss [38]. However, serum adiponectin levels were not affected by DEP-1 ASO treatment in our study. Interestingly, Christiansen et al. [39] showed that diet-induced weight loss enhanced circulating adiponectin, although exercise connected weight loss improved insulin sensitivity without changes in adiponectin level. Furthermore, possibly the extent and/or the time period of weight loss were not sufficient to produce a detectable reduction in adiponectin levels [40,41]. In addition, weight loss in our model maybe in part related to changes in thermogenesis, suggested by animal temperature differences assessed by LabMaster analyses, or impact on genes involved in lipogenesis.

Targeting DEP-1 by ASOs improved not only systemic effects including insulin sensitivity, but also insulin action at cellular/tissue level in insulin signaling pathways. Insulin-induced insulin receptor phosphorylation at tyrosine residues, and phosphorylation of downstream key components Akt Ser 473 and Akt Thr 308 was enhanced in liver, providing an additional molecular evidence for the increased insulin sensitivity *in vivo*. These data imply that DEP-1 acts as a regulator of the insulin pathway through dephosphorylation of the insulin receptor. However, we can not exclude whether further substrates of DEP-1 in the insulin signaling pathway exist, facilitating the enhanced signaling cascade [29,30]. Using the proximity ligation assay we unraveled the recruitment of DEP-1 into close proximity of the phosphorylated insulin receptor. In addition to the enhanced phosphorylation of the insulin receptor upon DEP-1 ASO treatment, these results indicate that the phosphorylated insulin receptor may serve as a substrate for DEP-1. This is also implicated by showing dephosphorylation of the insulin induced tyrosine-phosphorylated insulin receptor by recombinant DEP-1. However, no clear evidence for site-selectivity of DEP-1 regarding the insulin receptor was implicated *in vitro* (data not shown). This is in line with the observation by Barr et al. [42], showing that DEP-1, in contrast to other PTPs, is characterized by rather low *in vitro* substrate specificity.

It is important to emphasize that this study identified DEP-1 acting as a negative regulator in insulin signaling. However, the role of DEP-1 as a tumor suppressor in a number of epithelial cancers may not be neglected [19,43]. Future experiments are required to delineate if rather a tissue specific role of DEP-1 in metabolic tissues regarding insulin/glucose/lipid metabolism is required to improve safety. This may facilitate targeting DEP-1 in metabolic diseases without impairment of DEP-1 as a tumor suppressor protein, although neither spontaneous tumors were observed during DEP-1 ASO treatment in this study nor were described for DEP-1 KO mice [44].

## Conclusions

To conclude, our results indicate for the first time that targeting DEP-1 by using ASOs in mice exerts beneficial metabolic effects, in particular with regard to the role in insulin sensitivity and signaling. As a novel identified negative regulator of insulin receptor signaling, DEP-1 represents a potential target for the treatment of insulin resistance and type 2 diabetes.

## Methods

### Animal model

Male C57BL/6J mice were purchased from Janvier (Le Genest-Saint-Isle, France) at an age of 4 to 6 weeks. Mice were housed at room temperature (25°C) with a 12 hours light/dark cycle and fed for 10 weeks ad libitum with a low-fat diet (LFD) (n = 10) (10% kcal from fat; Altromin, Lage, Germany) or high-fat diet HFD (n = 33) (60% kcal from fat; Altromin, Lage, Germany). Afterwards mice of both groups (LFD n = 10, HFD n = 15) were treated with vehicle intraperitoneally for 6 weeks. Additional HFD mice (n = 18) were randomly assigned in a therapeutic approach to ASO treatment with control ASOs 5′-CCTTCCCTGAAGGTTCCTCC-3′ (n = 8) (ISIS 141923) and DEP-1 ASOs 5′-TACATTGCTGCCATCTCCAG-3′ (n = 10) (ISIS 285564) by injection intraperitoneally twice a week for 6 weeks at a dose of 25 mg/kg body weight. The used DEP-1 ASO sequence (ISIS 285564) was chosen based on initial ASO efficacy- and comparison experiments in C57BL/6J mice. Noteworthy, a chemically identical compound ASO (ISIS 141923) without known complementarity to any known gene sequence was used as a control ASO. Both ASOs were synthesized as 20-base phosphorothioate chimeric ASOs, where bases 1–5 and 16–20 had 2′-O-(2-methoxyethyl) modification. Before animals were sacrificed insulin (10 U/kg) (Insuman® Rapid, Sanofi Aventis, Berlin, Germany) was injected intraveneously and allowed to circulate for 2 min. Mice were killed under isoflurane anesthesia and organs were excised, weighed, and shock-frozen in liquid nitrogen and stored at −80°C until further use.

All animal procedures were in accordance with institutional guidelines and were approved by the Landesamt für Gesundheit und Soziales (LaGeSo, Berlin, Germany).

### Metabolic phenotyping (body weight, LabMaster, GTT, ITT, ELISA)

Body weight of all mice was measured twice a week throughout the whole study. Food and water intake, respiratory exchange ratio (RER), cage temperature, and locomotor activity were measured using an indirect calorimetry system (LabMaster, TSE Systems GmbH; Bad Homburg, Germany). Mice were placed in the calorimetry systems for up to 24 hours. After adaptation periods continuous measurements over 18 hours were used for analysis. An intraperitoneal insulin tolerance test (ITT) using a dose of 0.5 U/kg insulin (Insuman® Rapid, Sanofi Aventis, Berlin, Germany) and a glucose tolerance test (GTT) by intraperitoneal injection of 1 g/kg glucose (Glucosteril, Fresenius, Bad Homburg, Germany) were carried out in fasted mice. Tail vein blood was used for measuring the glucose concentration with a glucometer (Precision Xceed, Abbott, Wiesbaden, Germany) at different time points. Blood from mice was used to measure fasted serum insulin, leptin and adiponectin concentration by ELISA according the manufacturer’s instructions (Millipore, Schwalbach, Germany).

### Protein-tyrosine-phosphatase activity

Metabolic tissues (liver, gastrocnemius skeletal muscle, epididymal adipose fat tissue) were lysed in a dounce homogenizer using lysis buffer adjusted to tissue weights (150 mM NaCl, 25 mM C_2_H_3_NaO_2_, 1% NP-40, 10 mM DTT, aprotinin (4 μg/ml)). Pan-PTP activity measurements were performed by using crude cell lysate and reaction buffer (25 mM imidazole pH 7.4, 10 mM DTT) in a total volume of 50 μl, and DTT was added to a final concentration of 50 mM. For immunoprecipitation PTP analyses anti-DEP-1 and anti-PTP1B (AF1934 and AF3954 R&D Systems, Wiesbaden, Germany) were used at a concentration of 1 μg in an end-over-end reaction at 4°C over night. Immunoprecipitates were collected by Dynabeads® Protein G (Invitrogen, Karlsruhe, Germany) for 1 hour. After washing two times with lysis buffer and once with reaction buffer the precipitates were resuspended in 50 μl reaction buffer, and DTT was added to a final concentration of 50 mM. Pan-PTP activity and specific PTP activity was determined using a ^32^P-labeled src-optimal peptide as substrate. Measurements were performed in duplicate, and phosphatase activity was determined as the amount of ^32^P-labeled radioactivity released from the peptide after 7 min of incubation at 30°C.

### Quantitative real-time PCR

RNA was isolated with RNeasy Mini Kit (Qiagen, Hilden, Germany) following the manufacturer’s instruction for purification from cells and tissue (liver, skeletal muscle, adipose tissue), and cDNA synthesis was done with SuperScript®II (Invitrogen, Karlsruhe, Germany). Quantitative real-time PCRs were performed with SybrGreen (Applied Biosystems, Darmstadt, Germany) in duplicate and the expression of analyzed genes was normalized to the average expression of the housekeeping gene 18S. Following primer sequences (at final concentrations of 100 nM) were used for gene expression analysis: 18S fwd: 5′-GACTCTTTCGAGGCCCTGTA-3′ and rev: 5′-CACCAGACTTGCCCTCCAAT-3′; Insulin receptor fwd: 5′-CAATGGGACCACTGTATGCATCT-3′ and rev: 5′-ACTCGTCCGGCACGTACAC-3′; DEP-1 fwd: 5′-GCAGTGTTTGGATGTATCTTT-3′ and rev: 5′-CTTCATTATTCTTGGCATCTGT-3′; PTP1B fwd: 5′-CGGGAGGTCAGGGACCTT-3′ and rev: 5′-GGGTCTTTCCTCTTGTCCATCA-3′.

### Immunoblotting

Protein lysates from tissues were prepared using lysis buffer (150 mM NaCl, 20 mM Tris pH 7.5, 10 mM EDTA, 30 mM Sodiumpyrophosphat, 0.5% Deoxycholic acid, 0.5% Triton X-100; pH 7.5) supplemented with sodium vanadate (1 mM), PMSF (1 mM), and protease inhibitor cocktail tablets (Roche, Penzberg, Germany) and quantified using BCA Protein Assay Reagent (Thermo Fisher Scientific, Bonn, Germany). Precipitation of DEP-1 in liver protein lysates were performed by incubation with 40 μl of WGA (wheat germ agglutinin) for 4 hours, followed by three washing steps with lysis buffer. The precipitates were dissolved in SDS sample buffer.

Immunoblotting was performed according standard protocols with primary antibodies (DEP-1 (AF1934 R&D Systems, Wiesbaden, Germany), anti-phospho Akt (Ser 473), anti-phospho Akt (Thr 308), anti-pan Akt and anti-insulin receptor (4B8, Cell Signaling/New England Biolabs, Frankfurt a.M., Germany), anti-phospho Tyr (PY 99) (Santa Cruz, CA, USA), anti-phospho Tyr (4G10) and anti-GAPDH (Millipore, Schwalbach, Germany)). Horseradish peroxidase-conjugated anti-mouse (Dako, Hamburg, Germany) and anti-rabbit (GE Healthcare, Uppsala, Sweden) were used as secondary antibodies, and chemiluminescence (GE Healthcare, Uppsala, Sweden) served for visualization. Densitometric analyses were performed using ImageJ software.

### Proximity ligation assay (PLA)

Paraffin-embedded 5 μm liver sections from C57BL/6 mice were placed on SuperFrost Plus slides (Langenbrinck, Emmendingen, Germany) and fixed by heating in an incubator for 1.5 h at 60°C. To enable the antibodies to bind to proteins sections were deparaffinized, rehydrated and boiled 2 × 5 min in antigen retrieval buffer (citrate pH 6) in a microwave at 450 W. After cooling down at room temperature for 30 min the slides were blocked with 5% BSA in TBS-Tween 0.1% (TBS-T). As primary antibodies rabbit anti-insulin receptor antibody (ab5500, Abcam, Cambridge, UK; 1:100) was used together with either a mouse anti-pY100 antibody (Cell Signaling/New England Biolabs, Frankfurt a.M., Germany; 1:1200) or a goat anti-DEP-1 (AF1934, R&D systems, Wiesbaden, Germany; 1:1000) diluted in 5% BSA/TBS-T. The tissue was incubated with both antibodies at 4°C over night. The next day the tissue was washed 2 × 5 min in TBS-T and incubated with PLA probes (Olink, Duolink In Situ, Uppsala, Sweden) anti-rabbit MINUS and either anti-mouse PLUS or anti-goat PLUS, diluted in 5% BSA/TBS-T, for 1 h at 37°C. Before adding the diluted ligation mixture (Olink, Duolink In Situ Detection Reagents Orange) and incubating the tissue sections for 30 min at 37°C the slides were washed twice in TBS-T for 5 min. Afterwards the slides were washed 2 × 2 min in 1× Wash Buffer A. The tissue was then incubated with the diluted amplification mixture (In Situ Detection Reagents) for amplification by replicating the DNA circles via rolling circle amplification, for 100 min at 37°C and washed with 1× Wash Buffer B (2 × 10 min) and 0.01× Wash Buffer B (1 min). The stained tissue sections were mounted with Duolink In Situ Mounting Medium containing DAPI to stain the nuclei and stored at -20°C.

Pictures of the tissue sections were taken by using an epifluorescence microscope (Keyence, BZ-9000, Neu-Isenburg, Germany) with filters for visualization of DAPI and TRITC and a × 40 objective (CFI Plan Apo), and rolling circle products (RCPs) were counted. The collected pictures were analyzed using the BZ Analyzer software from Keyence. For clarity in printing, images shown where processed using an image editing software (ImageJ) where a maximum filter was applied to the PLA channel.

### Cell culture and insulin receptor dephosphorylation with recombinant proteins

AML12 liver cells were purchased from American Type Culture Collection (ATCC®, Wesel, Germany) and maintained in DMEM/F12, 10% FBS and 1% penicillin/streptomycin at 37°C in an atmosphere of 95% air and 5% CO_2_.

For insulin stimulation cells were starved over night followed by adding insulin (100 nM) for 10 min. Prepared protein lysates from unstimulated and stimulated cells were used for immunoprecipitation with insulin receptor antibody (4B8, Cell Signaling/New England Biolabs, Frankfurt a.M., Germany), by over night incubation. Immunoprecipitates were collected by Dynabeads® Protein G (Invitrogen, Karlsruhe, Germany) for 1 hour. After washing two times with protein lysis buffer and once with PTP reaction buffer the precipitates were resuspended in 40 μl PTP reaction buffer. Recombinant proteins DEP-1 and PTP1B (Abcam, Cambridge, UK) were added in a concentration of 1 μg followed by 30 min incubation at 30°C. Control reaction samples were treated with sodium vanadate 1 mM before recombinant proteins were added for dephosphorylation. The reaction was stopped by SDS sample buffer and boiling at 95°C for 3 min. Samples were then analyzed by immunoblotting with antibodies as indicated.

### Statistical analysis

Statistical differences between the groups were determined using unpaired Student’s *t* tests. The results are expressed as mean values ± standard error of the mean, and statistical significance was designated at *P* < 0.05.

## Competing interests

SB is employed by Isis Pharmaceutical, Inc.

## Authors’ contributions

JK and KK researched data, wrote, reviewed, and edited the manuscript. MT, MD, PS, HM, and CB researched data and assisted with the drafting of the paper. SB, AÖ, and UK reviewed and edited the manuscript, and contributed to discussion. All authors read and approved the final manuscript.

## Supplementary Material

Additional file 1: Figure S1 Analysis of tyrosine phosphorylation levels in untreated- and ASO treated mice in liver tissue. A: Immunoblotting analysis of tyrosine phosphorylation was performed in liver tissue derived from untreated (high-fat diet, HFD) and HFD-fed ASO treated mice (control ASO and DEP-1 ASO) using the monoclonal antibody pTyr 99. B: Quantification of phosphotyrosine-containing proteins was done after normalization to GAPDH and is expressed as arbitrary units. Densitometric analysis was performed using ImageJ software. Click here for file
