# Supplementary material for: Targeting density-enhanced phosphatase-1 (DEP-1) with antisense oligonucleotides improves the metabolic phenotype in high-fat diet-fed mice
Source: Cell Commun Signal. 2013 Jul 26;11:49. doi: 10.1186/1478-811X-11-49 (PMC3734182; doi:10.1186/1478-811X-11-49)
Supplement: Additional file 1: Figure S1 — Analysis of tyrosine phosphorylation levels in untreated- and ASO treated mice in liver tissue. A: Immunoblotting analysis of tyrosine phosphorylation was performed in liver tissue derived from untreated (high-fat diet, HFD) and HFD-fed ASO treated mice (control ASO and DEP-1 ASO) using the monoclonal antibody pTyr 99. B: Quantification of phosphotyrosine-containing proteins was done after normalization to GAPDH and is expressed as arbitrary units. Densitometric analysis was performed using ImageJ software. [file 1478-811X-11-49-S1.ppt]

## Slide 1
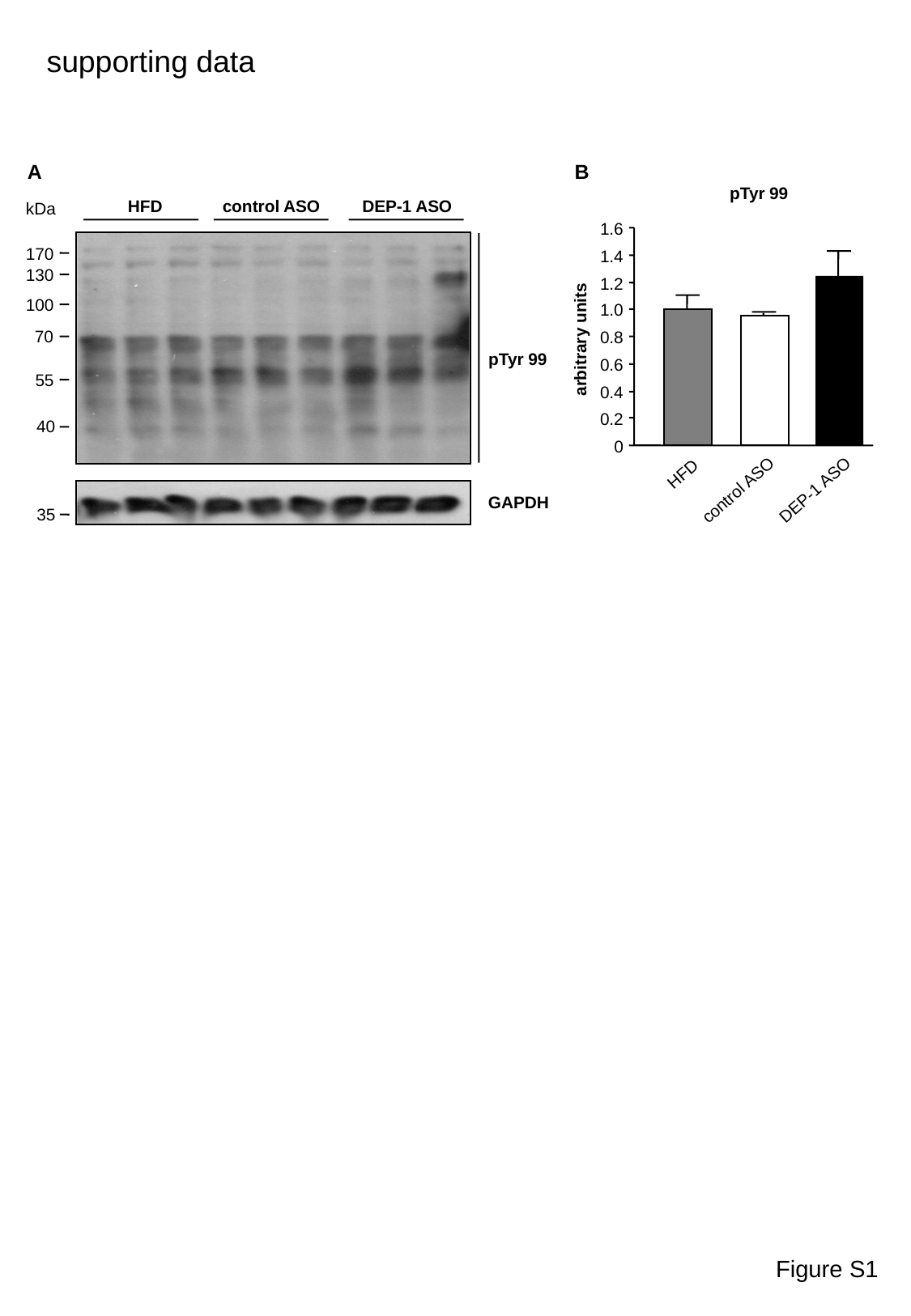

supporting data
A
B
pTyr 99
HFD
control ASO
DEP-1 ASO
kDa
1.6
170
1.4
130
1.2
100
1.0
70
0.8
arbitrary units
pTyr 99
0.6
55
0.4
0.2
40
0
HFD
DEP-1 ASO
control ASO
GAPDH
35
Figure S1
